# Identifying the content and context of pain within paediatric rheumatology healthcare professional curricula in the UK: a summative content analysis

**DOI:** 10.1186/s12969-021-00614-1

**Published:** 2021-08-21

**Authors:** Rebecca Rachael Lee, Janet E. McDonagh, Mark Connelly, Sarah Peters, Lis Cordingley

**Affiliations:** 1 Centre for Epidemiology Versus Arthritis, Centre for Musculoskeletal Research, Division of Musculoskeletal and Dermatological Sciences, Faculty of Biology, Medicine and Health, Manchester Academic Health Science Centre, University of Manchester, Manchester, UK; 2 National Institute for Health Research Biomedical Research Centre, Manchester University Hospital NHS Trust, Stopford Building, Oxford Road, Manchester, M13 9PT UK; 3 Royal Manchester Children’s Hospital, Manchester University Foundation Hospitals Trust, Manchester, UK; 4 Children’s Mercy Kansas City, 2401 Gillham Road, Kansas City, USA; 5 Manchester Centre for Health Psychology, Division of Psychology and Mental Health, University of Manchester, Manchester, UK

**Keywords:** Pain, Healthcare professionals, Paediatric rheumatology, Training, Curricula, Competencies, Assessment, Management, Communication

## Abstract

**Background:**

The curriculum for professionals working in paediatric rheumatology should include pain but it is unclear to what extent this currently occurs. The aim of this study was to identify pain-related curriculum content and the context in which pain is presented in educational and training documentation for healthcare professionals in this clinical speciality.

**Methods:**

Core curricula documents from UK based professional organisations were identified in partnership with healthcare professionals. Documents were analysed using a summative content analysis approach. Key pain terms were quantified and weighted frequencies were used to explore narrative pain themes. Latent content was interpreted qualitatively to explore the context within which pain terms were positioned.

**Results:**

Nine curriculum documents were identified and analysed from doctors, nurses, physiotherapists and occupational therapists specialising in paediatric rheumatology. Pain themes represented a mean percentage of 1.51% of text across all documents. Pain was rarely presented in the context of both inflammatory and non-inflammatory condition types despite being a common feature of each. Musculoskeletal pain was portrayed simply as a ‘somatic’ symptom, rather than as a complex phenomenon involving biological and psychosocial processes. Content around the assessment and management of pain was vague and inexplicit.

**Conclusion:**

Current educational and training documentation in paediatric rheumatology do not include core pain topics. Curricula for these healthcare professionals would benefit from updates in contemporary pain theories and examples of in-context, evidence-based pain practices. This should be a priority starting point for optimising patient pain care in paediatric musculoskeletal healthcare.

## Background

Chronic musculoskeletal pain conditions are amongst the leading cause of disability globally and consequently one of the largest economic health burdens [1–3]. Children and young people are referred to paediatric rheumatology clinics with a wide spectrum of pain conditions and musculoskeletal diseases that have pain as a chief complaint [4–6]. In particular, chronic musculoskeletal pain is a common feature and presentation in the context of both inflammatory diseases such as juvenile idiopathic arthritis [7] and non-inflammatory conditions such as back pain [8]. Left unmanaged, pain occurring as a child or young person can continue into early adulthood in many conditions [9, 10]. Therefore, pain assessment and management are critical components for those effectively working with all children and young people presenting to paediatric rheumatology practices [11–14].

Comprehensive assessment of pain is important for informing healthcare professionals’ strategies for developing and tailoring personalised pain management plans [15]. Furthermore, healthcare professionals’ recognition of and empathetic or compassionate attitudes towards pain are crucial for children and young people’s well-being even when the targeted outcome may not be to ‘cure’ pain [16, 17]. Several guidelines for pain assessment and management have been published in recent years [14, 18]. In order to follow these guidelines and to effectively assess, communicate about, and manage pain, healthcare professionals require an in-depth understanding of pain processes based on core knowledge and skills. A core pain curriculum for pain published by The International Association for the Study of Pain (IASP) [19–21] states that education on the multi-dimensionality nature of pain and training in pain assessment and its management should be provided as a minimum to all healthcare professionals.

Research exploring the extent to which pain is covered in the undergraduate, or pre-registration curricula of healthcare professionals in the UK has found that it is limited. For example, physiotherapy students on average receive three times the amount of pain education of medical students (38 h versus 13 h) [22]. For paediatric nursing students, the average number of pain education hours is 10 and for occupational therapy students the average time spent covering pain content is 14 h. Similar conclusions on the lack of pain specific healthcare professional education and training are echoed internationally and across all healthcare professions [23–27].

Limited pain coverage at undergraduate level for healthcare professionals could potentially be offset by more comprehensive pain education and training when professionals begin to specialise in their respective disciplines. The postgraduate clinical training curricula for doctors in rheumatology has undergone substantial reform in the last decade [28]. However, the broader multi-disciplinary team of healthcare professionals in paediatric rheumatology continue to report limited pain education and training [29]. Consequently, some paediatric rheumatology healthcare professionals report low confidence in initiating or engaging in conversations about pain with children or young people who come to clinic.

To date, no studies have systematically investigated gaps and opportunities to improve education and training in the clinical speciality of paediatric rheumatology, particularly with regards to pain. This study aimed to explore the extent to which pain content is included in the contemporary curricula guiding the postgraduate training of healthcare professionals in paediatric rheumatology, and the context within which pain content is currently presented.

## Methods

### Study design

A directed search of the main UK organisations associated with the accreditation of healthcare professionals was performed; doctors (Royal College of Paediatric and Child Health [RCPCH]), nurses (Royal College of Nursing [RCN]), physiotherapists (Chartered Society of Physiotherapy), occupational therapists (Royal College of Occupational Therapists) and psychologists (British Psychological Society). Following this, a systematic hand search of the grey literature and documental data was conducted by entering key search terms into the most widely used web search engines in the UK; Google, Microsoft Bing and Yahoo. Key terms included ‘education’, ‘training’, ‘curriculum’, ‘curricula’, ‘competency’, ‘competencies’, ‘syllabus’, ‘syllabi’, ‘roles’, ‘responsibilities’, and ‘frameworks,’ in combination with the terms ‘paediatric’, ‘adolescent’ and ‘rheumatology’. Key healthcare professional stakeholders across the UK (*N* = 10) were directly approached by the research team to help with identifying documents. One UK paediatric rheumatologist trainee group (*N* = 72 members) and one national UK clinical nurse specialist group (*N* = 77 members) (both affiliated with The British Society for Rheumatology) were also approached.

### Materials

For the purposes of this study, documents were considered to be part of the core curricula if they were used to guide the education and/or the training of healthcare professionals specifically in the clinical speciality of paediatric rheumatology. Documents dated from January 2010 onwards were eligible for review. Document searches were conducted between June and November 2020 and those fitting specific inclusion and exclusion criteria were included for review (see Table 1).

**Table 1:** Inclusion and exclusion criteria for review of curricula in paediatric rheumatology

Category	Inclusion	Exclusion
Targeted professional group	• Consultant paediatric rheumatologists • General paediatricians • Trainee rheumatologists • Nurses • Allied health professionals (physiotherapists and/or occupational therapists) • Psychologists	• Professional groups managing symptoms that are not musculoskeletal (e.g. ophthalmologists)
Targeted patient group	• Chronic musculoskeletal conditions managed in paediatric and adolescent rheumatology	Documents which are: • Adult rheumatology • Pain service/team specific • Generic paediatric conditions (e.g. ‘complex health needs’) • Management of features outside of the musculoskeletal system (e.g. uveitis)
Organisation/document authors	• UK based professional organisations and networks with a focus on care specifically in paediatric rheumatology	• Outside of the UK • Outdated documents
Document type/objective	• Curriculum/curricula • Syllabus/syllabi • Competency/competencies frameworks • Roles and responsibilities of professionals	• Undergraduate documents • Educational resources without an underlying curricula component to be reviewed • Research articles

### Analysis

Once identified, all documents were analysed in NVivo version 12 (QSR International, Warrington, UK) as textual data. A summative content analysis approach was adopted for data analysis [30, 31]. The method allows for the creation of a coding framework for concepts of interest, which can be used to systematically and transparently identify the frequency of key components of documents. The summative content analysis method also includes qualitative analysis of underlying themes identified in the quantitative analysis.

The analysis began with the development of a coding framework. The coding framework was created through an iterative process of both top-down coding of pre-identified key pain words (e.g., ‘pain’, ‘pain assessment’, ‘pain communication’, ‘pain measurement’) and a bottom-up process of additional terms identified during the analysis (e.g., ‘painful’ and ‘pain control’). The coding framework continued to be refined until consensus was reached among members of the research team. The coding framework was used to identify and quantify key pain terms. It was applied flexibly to allow for terms used separately such as ‘pain’ and ‘management’ within the same sentence to be coded as ‘pain-management’ if that was the context within which the terms were used. Weighted frequencies of pain themes used across the texts was calculated by coding whole sections focused upon pain specific content, relative to the whole amount of ‘other’ text in the documents.

In the qualitative summative content analysis, data can either be interpreted through a latent approach or a semantic approach. Latent content analysis is more interpretative, exploring the implied and embedded meaning within text, whereas a semantic approach is where the researcher does not go beyond what participants have said, with very limited use of interpretation in development of any themes [31–34]. In this study, the context within which key terms and phrases appeared was explored in a latent content analysis. This meant that the inherent meaning underlying the use of the pain terminology and phrases within the documents could be explored. Pain terms and phrases were interrogated through the active use of schemas and theory to make sense of them within their context in documents and their tacit implications for practice. In order to perform this part of the analysis, the researcher coded sections of the documents into overarching interpretative themes and subthemes using NVivo. Findings from the latent content analysis are presented as narrative themes.

## Results

Nine documents fitting the inclusion criteria were identified. Five documents were created for doctors. Three of these were linked syllabi used to guide the training of doctors into practice in general paediatrics, with each syllabus including additional content to support progression from Level 1 to 3. One competency document was specifically created to shape the training of doctors specialising in paediatric rheumatology and one competency framework was used to guide the training of general paediatricians with a special interest in paediatric rheumatology. Three nursing documents were identified, with two of these focusing on the competencies of nurses and one focusing on roles and responsibilities. One document was identified for directing the competencies of allied health professionals (applicable to physiotherapists and occupational therapists only) Table 2.

**Table 2:** Frequencies of key terms in UK curricula and competencies in paediatric rheumatology speciality

***Target healthcare professional***	***Document title***	***Document authors/organisations***	***Length of document***	***Objective of the document***	***Total key terms identified***	***Weighted percentage of ‘pain’ phrases within document***	***Pain or pains***	***Painful***	***Pain assessment/measurement***	***Pain communication***	***Pain control/medications***	***Pain management/treatment***
Doctors	Generic syllabus level 1.	The Royal College of Paediatrics and Child Health (RCPCH), 2018.	60 pages	“To train doctors to acquire detailed knowledge and understanding of health and illness in babies, children and young people.”	**10**	0.56%	7	0	0	0	0	3
Doctors	Generic syllabus level 2.	The Royal College of Paediatrics and Child Health (RCPCH), 2018.	54 pages	As above.	**8**	0.67%	5	0	1	0	0	2
Doctors	Generic syllabus level 3.	The Royal College of Paediatrics and Child Health (RCPCH), 2018. *To be implemented alongside the document below	40 pages	As above.	**1**	0.16%	0	0	0	0	0	1
Doctors	Paediatric rheumatology syllabus level 3.	The Royal College of Paediatrics and Child Health (RCPCH), 2018.	22 pages	As above.	**13**	2.5%	3	1	1	0	1	7
Doctors	A framework of competences for the special interest module in paediatric rheumatology	The Royal College of Paediatrics and Child Health (RCPCH), 2014.	14 pages	“It is for doctors at Level 3 in their General Paediatric training who wish to work towards an expertise in Paediatric Rheumatology during Level 3 training or post CCT.”	**8**	5.41%	7	0	0	1	0	0
Nurses	A competency framework for rheumatology nurses.	Royal College of Nursing (RCN), 2020. *To be implemented alongside the document below	37 pages	***“***This document provides a competency and role development framework for rheumatology practitioners in clinical practice most commonly termed as rheumatology nurse specialists (RNS).”	**5**	0.46%	1	0	1	0	0	3
Nurses	Core competencies for paediatric rheumatology clinical nurse specialists and advanced nurse practitioners. Administering disease modifying anti rheumatic drugs (DMARDs) and biologic therapies to children and young people with rheumatological conditions.	The British Society for Paediatric and Adolescent Rheumatology (BSPAR) Section Council Nurse Group, 2014.	6 pages	***“***The competency levels set out here will help to identify the range of skill mixes that can be used in providing CYP rheumatology care.”	**4** *Pain used in the context of assessment AND management therefore coded twice	1.22%	0	1	1	0	1	2
Nurses	Role of the paediatric rheumatology nurse.	Scottish paediatric and adolescent rheumatology network (SPARN), 2016.	1 page	Not reported.	**0**	0%	0	0	0	0	0	0
Allied Health Professionals	Paediatric rheumatology allied health professional competencies	The British Society for Paediatric and Adolescent Rheumatology (BSPAR),2019.	5 pages	“The following cover the recommended competencies for therapists (Occupational Therapists and Physiotherapists) involved in the care of children and young people with rheumatology conditions.”	**6**	2.63%	5	0	0	0	0	1

### Frequency of key terms

Within the nine documents reviewed, there were 55 occurrences related to pain terms, which represented a mean percentage of 1.51% of text across all documents. The majority of pain terms occurred in the doctors RCPCH Paediatric Rheumatology Syllabus Level 3 (*n* = 13, 23.64%), followed by the RCPCH Generic Syllabus Level 1 (*n* = 10, 18.18%). The RCPCH Generic Syllabus Level 2 and the RCPCH Framework of Competencies for Doctors Specialising in Paediatric Rheumatology had the same frequency of pain terms (*n* = 8, 14.55%). The RCPCH Framework of Competencies for Doctors Specialising in Paediatric Rheumatology also had the largest amount of pain related themes relative to other text incorporated into the curricula (5.41% of all text focused on pain). This demonstrates that across all healthcare professional documents, medical curricula included the most pain specific content. This was followed by the British Society for Paediatric and Adolescent Rheumatology (BSPAR) Allied Health Professional Competencies (*n* = 6, 10.91%) and the RCN Competency Framework for Rheumatology Nurses (*n* = 5, 9.09%). No pain terms were found within the Scottish Paediatric and Adolescent Rheumatology Network (SPARN) Role of the Paediatric Rheumatology Nurse. This document was therefore excluded from further qualitative analysis.

### Qualitative themes

#### Types of conditions

Together, the documents referred to a wide range of specific musculoskeletal pain conditions such as chronic regional pain syndrome, generalized idiopathic pain syndrome, pain amplification syndrome, growing pains, mechanical pains, biomechanical pains, over-use syndromes, back pain, abdominal pain, knee pain, referred pain and biomechanical pain. Two documents considered the importance of acute pain in practice, encouraging nurses to identify and manage (RCN Competency Framework for Rheumatology Nurses) and doctors to understand sedation for painful procedures (RCPCH Paediatric Rheumatology Syllabus Level 3). Two of the documents aimed at doctors (RCPCH Generic Syllabi Level 1 and 2) discussed the importance of pain in the context of palliative care.

#### Pain features

None of the documents identified which features and characteristics of pain are of particular importance. For example, there was no reference to pain intensity, location, emotion/affect or interference. The BSPAR Competencies for Paediatric Rheumatology Clinical Nurse Specialists and the RCPCH Generic Syllabus Level 2 for doctors highlighted that these professionals should be aware of how to manage ‘painful joints’. In the RCPCH Framework of Competencies for Doctors Specialising in Paediatric Rheumatology, ‘joint’ pain and ‘muscle’ pain were specified.

#### Identifying and managing pain symptoms in inflammatory and non-inflammatory conditions

There was a clear distinction between inflammatory and non-inflammatory pain in terms of identifying and managing the causes of pain, with many of the documents tending to consider pain in the context of only one of these pain types. Two of the documents considered causes of pain from an inflammatory pathology perspective such as inflammatory causes of joint and back pain (RCPCH Framework of Competencies for Doctors Specialising in Paediatric Rheumatology) and arthritis (BSPAR Allied Health Professional Competencies). Content on the management of pain symptoms primarily focused on non-inflammatory musculoskeletal conditions such as hypermobility, growing pains, back pain and pain amplification syndrome in the RCPCH Paediatric Rheumatology Syllabus Level 3. The BSPAR Competencies for Paediatric Rheumatology Clinical Nurse Specialists document specifically developed for guidance around the administration of disease modifying anti-rheumatic drugs and biologic therapies highlighted that pain should be managed within the context of chronic inflammatory disease.

The RCPCH Generic Syllabus Level 1 and the RCPCH Framework of Competencies for Doctors Specialising in Paediatric Rheumatology were the only documents to highlight the importance of pain management as a feature of both inflammatory and non-inflammatory conditions.

#### Understanding pain and diagnoses

The BSPAR Allied Health Professional Competencies emphasised the need to consider the biopsychosocial model approach in recognising pain. This approach was positioned as particularly relevant for interpreting the impact of pain. In terms of doctors training on the understanding of pain, skills in differential diagnostics around pain (mechanical pain, joint pain, back pain, growing pains and marked musculoskeletal pain) appeared to be prioritised. The RCPCH Generic Syllabus Level 2, RCPCH Paediatric Rheumatology Syllabus Level 3 and the RCPCH Framework of Competencies for Doctors Specialising in Paediatric Rheumatology directed doctors understanding towards the associations between chronic pain and fatigue. The RCPCH Generic Syllabus Level 2 stated that the professional should be able to explain how pain syndromes can present with musculoskeletal symptoms and that they should also be able to interpret ‘normal’, ‘abnormal’ or ‘unusual’ common ‘somatic’ musculoskeletal pain symptoms.

#### Assessment of pain and developmental appropriateness

Two documents explicitly referenced effective evaluation and assessment of musculoskeletal pain (RCPCH Paediatric Rheumatology Syllabus Level 3 and the RCPCH Generic Syllabus Level 2). One document identified that an awareness of assessment options was necessary (BSPAR Competencies for Paediatric Rheumatology Clinical Nurse Specialists). The RCN Specialist Nurse Competencies was the only document to unequivocally state that the use of age-appropriate pain assessment tools was key in practice, taking into account the developmental appropriateness of such tools. One of the documents discussed the importance of being able to respond to children and young people that could not verbally express their pain (RCPCH Generic Syllabus Level 1).

#### Communication of pain

The RCPCH Framework of Competencies for Doctors Specialising in Paediatric Rheumatology was the only document to explicitly mention communication issues in the context of pain (mechanical pain in particular). The main body of this curricula text included reference to trainees having skills in how to ‘counsel’ children, adolescents and their parents about chronic pain. However, in the assessment criteria, pain communication specifically with parents and carers appeared to be emphasised as an essential skill, rather than knowing how best to involve children and young people directly when communicating about or assessing their pain.

#### Pharmacological management of pain

Two documents referenced the pharmacological management of pain, with the BSPAR Competencies for Paediatric Rheumatology Clinical Nurse Specialists highlighting the importance of basic training in pain control and the RCPCH Paediatric Rheumatology Syllabus Level 3 stating that the trainee doctor should be able to initiate and monitor a range of drugs for pain control.

#### Non-pharmacological management of pain

The RCN Specialist Nurse Competencies referred to the implementation of both pharmacological and non-pharmacological management strategies for pain without explicit mention of what these strategies included. The BSPAR Competencies for Paediatric Rheumatology Clinical Nurse Specialists highlighted that an ‘awareness’ of therapeutic options for pain was important. Throughout all of the documents, there was little distinction in what aspects and outcomes of pain should be managed, apart from in the BSPAR Allied Health Professional Competencies which placed specific importance upon management of the complexities of pain impact.

#### Multi-disciplinary and inter-disciplinary pain management

The RCPCH Paediatric Rheumatology Syllabus Level 3 emphasised that the doctor should have the ability to co-ordinate a multi-disciplinary team approach to pain management. Many of the other documents focused on referral for management of persistent pain outside of the team to other ‘appropriate’ inter-disciplinary services such as the community, secondary care and/or other specialist paediatric teams (Framework of Competencies for Doctors Specialising in Paediatric Rheumatology, RCN Specialist Nurse Competencies and BSPAR Allied Health Professional Competencies). The RCPCH General Syllabus Level 3 suggested that the doctor should incorporate key aspects of management programmes into their care.

## Discussion

To our knowledge, this study provides the first in-depth evaluation of pain content within curricula guiding healthcare professionals training in the specialist area of paediatric rheumatology in the UK. There are several pertinent issues highlighted, predominantly emphasising limited coverage of pain within the identified curricula and conceptual or theoretical limitations where pain content is included. Such gaps are important to address considering that pain is common in children and young people with chronic musculoskeletal pain cared for within the specialty.

In the curricula identified, pain content was included primarily in the context of either non-inflammatory or inflammatory conditions, seldom in the context of both. Presenting pain content only in the context of inflammatory disease reinforces the idea that pain stems directly from disease processes, a medical model paradigm which is unhelpful for interpreting experiences of chronic pain [35]. However, presenting pain only in the context of non-inflammatory disease processes is also problematic as it suggests that pain is not a feature of inflammatory disease processes such as juvenile idiopathic arthritis. Pain management may be overlooked if the perceptions of healthcare professionals are focused upon contexts of pain limited to one type of condition.

Another conceptual issue uncovered was that of pain positioned predominantly as a ‘somatic’ symptom. The term ‘somatic’ unduly denotes that a feature of illness relates to the body and is a distinct experience from that of the mind. Coupling the term ‘somatic’ alongside pain suggests that pain is a purely biological process without psychological or social components. Pain may indeed commonly co-occur as a symptom of a broader ‘somatisation’ disorder, but pain is not exclusively somatic [36]. Referring to pain as a somatic symptom in curricula undermines complexities and may also contribute to outdated medical model perspectives. Despite several of the documents indicating an underlying medical model tone to the pain content provided, documents created for allied health professionals explicitly referenced the importance of developing an understanding of pain from a biopsychosocial perspective. This approach should be embedded across all healthcare professions [37].

We identified relative gaps in pain assessment content in particular. Where pain content was included in curricula, it was likely to be in the context of pain management rather than assessment. This limited focus upon pain assessment likely impedes a professionals’ ability to effectively manage pain as skills in assessment are a prerequisite for appropriate management. None of the documents included within the review referred to the salience of particular pain dimensions (e.g. intensity, location, qualities of pain), pieces of information that have been shown to be used differently in informing pain management decisions [38].

Pain management content provided across curricula and competency documents was found to be vague overall. For example, pharmacological and non-pharmacological approaches were mentioned as effective strategies but with no explicit detailing of what these therapeutic avenues may include. There also appeared to be a distinction between professionals’ *awareness* of approaches, with little explicit reference to *implementation* or *delivery* of such strategies. There was no reference to significant pain outcomes such as pain understanding and education, pain coping, or pain self-management, all of which are important features of effective management [39]. Several documents suggested that professionals should borrow key principles from management programmes outside of the paediatric rheumatology service. However, which management principles to adopt and from which programmes were not specified.

The IASP core curricula for pain serves as a helpful benchmark for healthcare professional education in pain and can help elucidate specific gaps in content. Counter to recommendations in the IASP core curricula, paediatric rheumatology curricula does not foster conceptualization of pain as a multi-dimensional experience encompassing physiological, sensory, affective, cognitive, behavioural, social and cultural mechanisms. The curricula and competencies reviewed also do not provide detail on the granularities of pain assessment practices and pain communication. For example, the documents neglect to describe the breadth of measurement tools available, gold standard assessment and limitations, capturing exhaustive pain histories, or knowledge of how to work collaboratively with other professionals to collect comprehensive pain reports. There is no content around pertinent pain management issues such as personalised goal-setting, long-term planning or training on how to approach broader treatment challenges such as patient motivation or anxieties.

Some of the gaps in pain content identified may be under-provided as a consequence of the need to keep curricula brief [40]. Similarly, the lack of content relating to specific applied issues such as pain communication may be due to the fact that developmentally appropriate general communication skills are core knowledge to all paediatric healthcare professionals [41]. Using pain as an example in these components of the curricula would enhance understanding and emphasise the importance of particular skills in this area.

### Future recommendations for curricula and competencies

Using the findings of this review and the IASP core curriculum, there are several recommendations for forthcoming training documents guiding this clinical speciality. Curricula and competencies should:

- Re-conceptualise pain as integral to both inflammatory and non-inflammatory disease processes.
- Increase understanding about pain as a biopsychosocial phenomenon.
- Be more balanced in terms of the breadth of pain assessment versus pain management content covered and more explicit in which particular techniques and strategies are important for implementation in both.
- Outline which pain assessment, pharmacological and non-pharmacological pain management options professionals should be aware of and clarify which of these specific options different professional groups should be skilled in performing.
- Put a greater emphasis upon the importance of pain communication and developmental considerations which would also ensure the appropriateness of techniques used within clinical practice.
- Clarify which key management programmes and principles professionals should incorporate into their practice, enabling professionals to access appropriate resources.
- Develop curricula and competencies for other professions that work alongside doctors, particularly for psychologists whose role in pain management can be essential in paediatric rheumatology.

In practice, these recommendations can be implemented in several ways. Pain should appear as a priority component in future revisions of curricula and outdated pain theory and concepts should be removed. These modifications should be publicised widely. It would also be useful to present updated curricula alongside opportunities for additional knowledge and skills training which makes explicit links to contemporary pain theory. Inviting experts working in the field of paediatric pain to identify, shape and review new content provided on pain would be valuable.

### Strengths and limitations

The active participation of healthcare professionals to identify documents for this review was an important strength of this research and this practitioner involvement is key to further identifying additional training opportunities accessed across the speciality both within and outside of the UK. During the process of identifying documents for this review, it was clear that there were further opportunities for healthcare professionals to improve their knowledge and skills in musculoskeletal medicine and pain [42]. However, there are several problems with the informalities of identifying extra-curricular activity. Unendorsed curricula make it difficult to quality assure education and training. Furthermore, a lack of funding available to support attendance (both to attend the course and to buy out clinical time of the professional) is a barrier affecting accessibility [43]. Pain should be core to paediatric rheumatology training globally and should not be optional or resource/funding dependant.

A limitation of the current study is that there were no key topic comparators included to contrast the level of pain content provided against. There may be little focus upon the theoretical frameworks, assessment, communication and/or management of other pertinent issues such as fatigue, disability or transition. Future development of curricula should take account of the balance of other primary concerns in musculoskeletal disease.

Lastly, the Anglocentric focus of this paper may limit the generalisability of these findings, however, it would be interesting to see a similar analysis of documents from different countries to explore how pain content and context compares to UK based training and competencies.

## Conclusions

This review has highlighted significant gaps in pain content and misrepresentations of pain mechanisms and context in the curricula of healthcare professionals in paediatric rheumatology in the UK. Findings suggest that some gaps are pertinent to particular professional groups. However, many of the conceptual problems in how pain content is portrayed are embedded in the curricula of all healthcare professions working in this speciality. Professionals would benefit from exposure to contemporary pain theory and incorporation of evidence-based pain practices. Addressing the absence of particular pain topics, improving the depth of pain content and updating the context of pain within curricula for rheumatology healthcare professionals is critical for ensuring comprehensive and quality pain care for children and young people managed in these centres.

## Data Availability

The datasets analysed during the current study are available in the Royal College of Paediatrics and Child Health (links 1–5), The Royal College of Nursing (link 6), The British Society for Paediatric and Adolescent Rheumatology (links 7 and 9) and The Scottish Paediatric and Adolescent Rheumatology Network repositories (link 8): 1. https://www.rcpch.ac.uk/sites/default/files/2018-03/rcpch_progress_curriculum_level_1_generic_syllabus_for_use_from_1_aug_2018.pdf 2. https://www.rcpch.ac.uk/sites/default/files/2020-03/Curriculum%20Level%202%20Generic%20Syllabus%20%28v1.2%29%20updated%2020200320_0.pdf 3. https://www.rcpch.ac.uk/sites/default/files/2021-03/Generic%20Syllabus%20-%20Level%203%20%28v1.1%29%20updated%2020210331_0.PDF 4. https://www.rcpch.ac.uk/sites/default/files/2018-03/paediatric_rheumatology_syllabus_final.pdf 5. https://www.rcpch.ac.uk/sites/default/files/2018-05/rheumatology_2.doc 6. https://www.rcn.org.uk/-/media/royal-college-of-nursing/documents/publications/2020/march/009-004.pdf?la=en 7. https://www.rheumatology.org.uk/Portals/0/Documents/Guidelines/Paediatric%20guidelines/Core_competencies_Paediatric_Rheumatology_Clinical_Nurse_Specialists_Advanced_Nurse_Practitioners.pdf 8. https://www.sparn.scot.nhs.uk/wp-content/uploads/2016/11/Role-of-Paediatric-Rheumatology-Nurse.pdf 9. https://www.rheumatology.org.uk/Portals/0/Documents/Guidelines/Paediatric%20guidelines/AHP_competencies_CYP_2019.pdf?ver=2019-07-01-152950-443

## Abbreviations

IASP: The International Association for the Study of Pain
RCPCH: The Royal College of Paediatrics and Child Health
RCN: The Royal College of Nursing
BSPAR: The British Society for Paediatric and Adolescent Rheumatology
SPARN: The Scottish Paediatric and Adolescent Rheumatology Network
